# Supplementary figures and images for: Lactobacillus rhamnosus GG improves cognitive impairments in mice with sepsis
Source: PeerJ. 2024 May 28;12:e17427. doi: 10.7717/peerj.17427 (PMC11141560; doi:10.7717/peerj.17427)

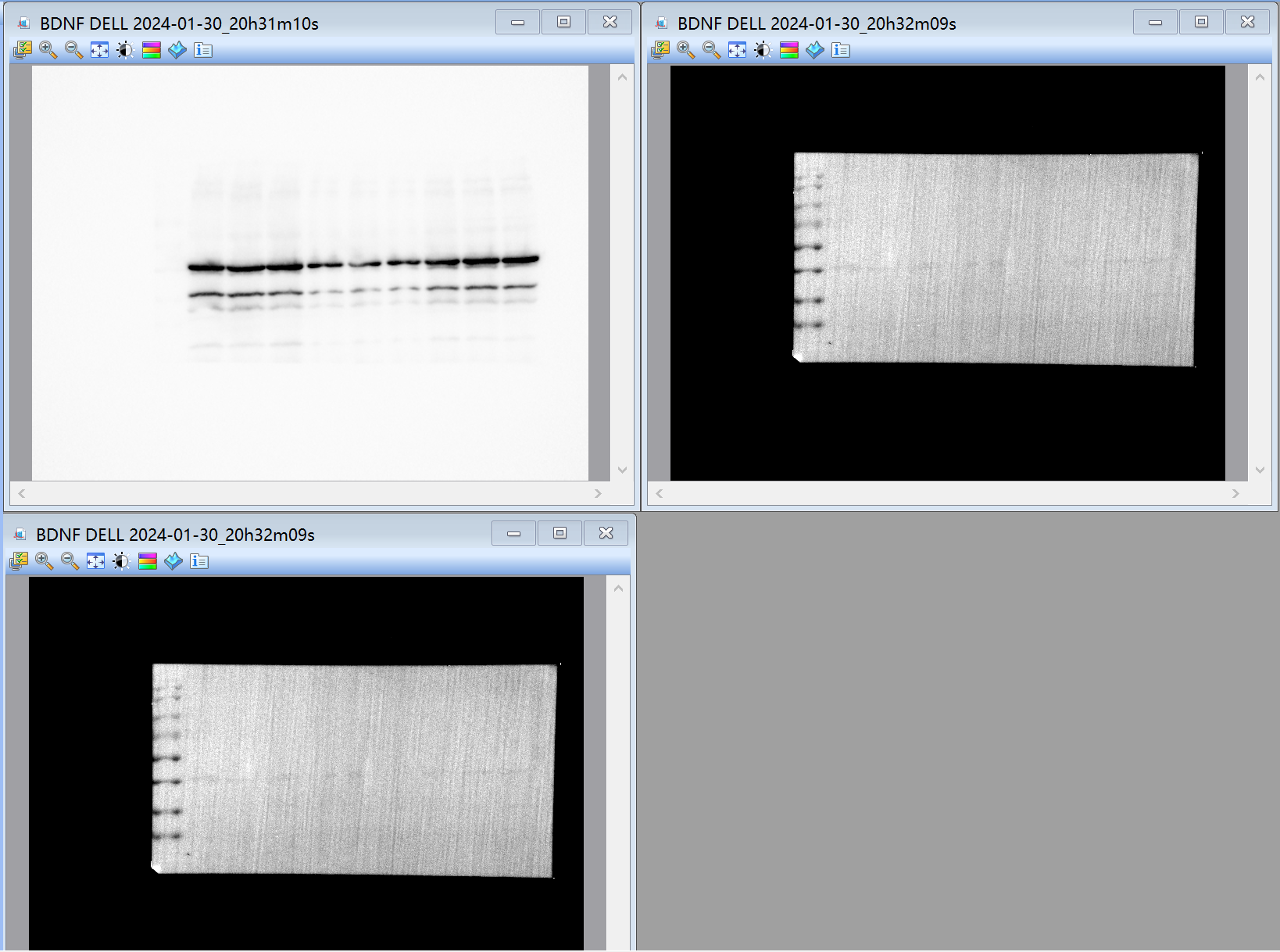

Supplement: Supplemental Information 2 — The raw data shows that the levels of BDNF expression and TrkB phosphorylation (p-TrkB) in the hippocampal region of the CLP mice were significantly reduced, relative to that in the sham group, but almost rescued in the LGG group of mice. [file peerj-12-17427-s002.zip › Supplementary files 2. (WB raw data)/BDNF.tiff]

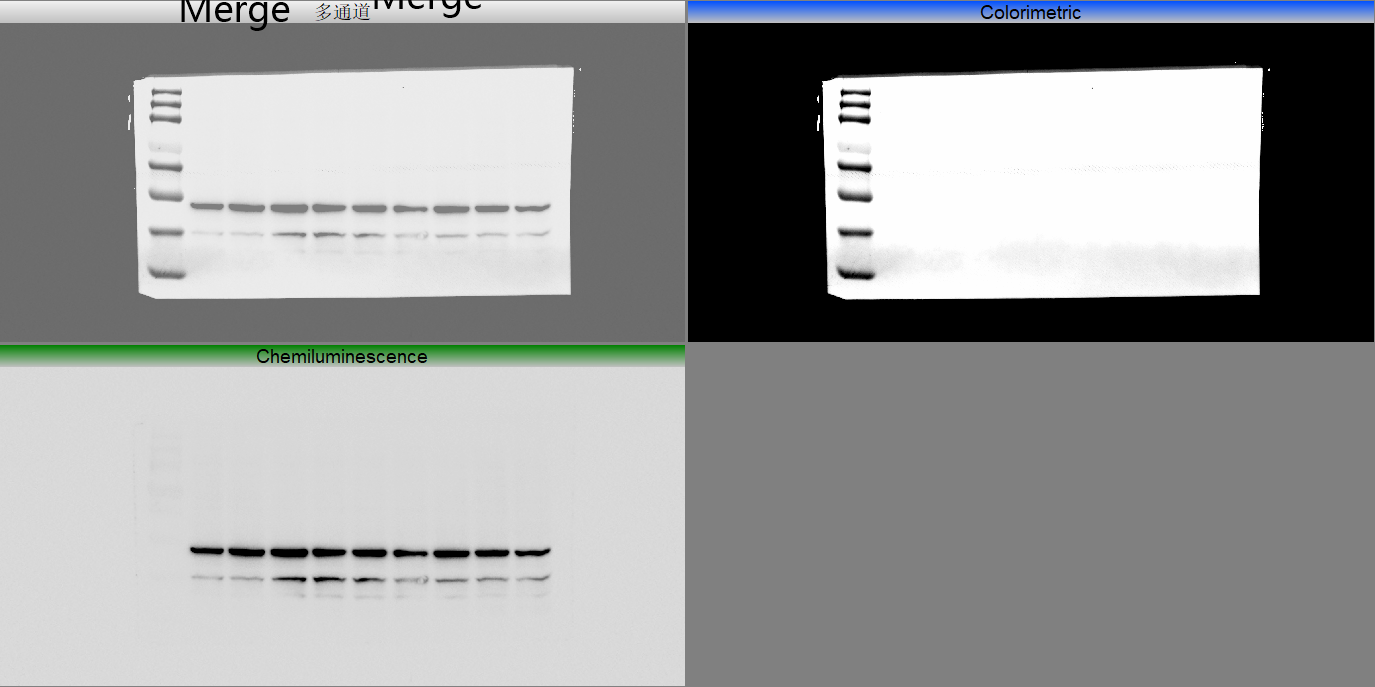

Supplement: Supplemental Information 2 — The raw data shows that the levels of BDNF expression and TrkB phosphorylation (p-TrkB) in the hippocampal region of the CLP mice were significantly reduced, relative to that in the sham group, but almost rescued in the LGG group of mice. [file peerj-12-17427-s002.zip › Supplementary files 2. (WB raw data)/BDNF1.tif]

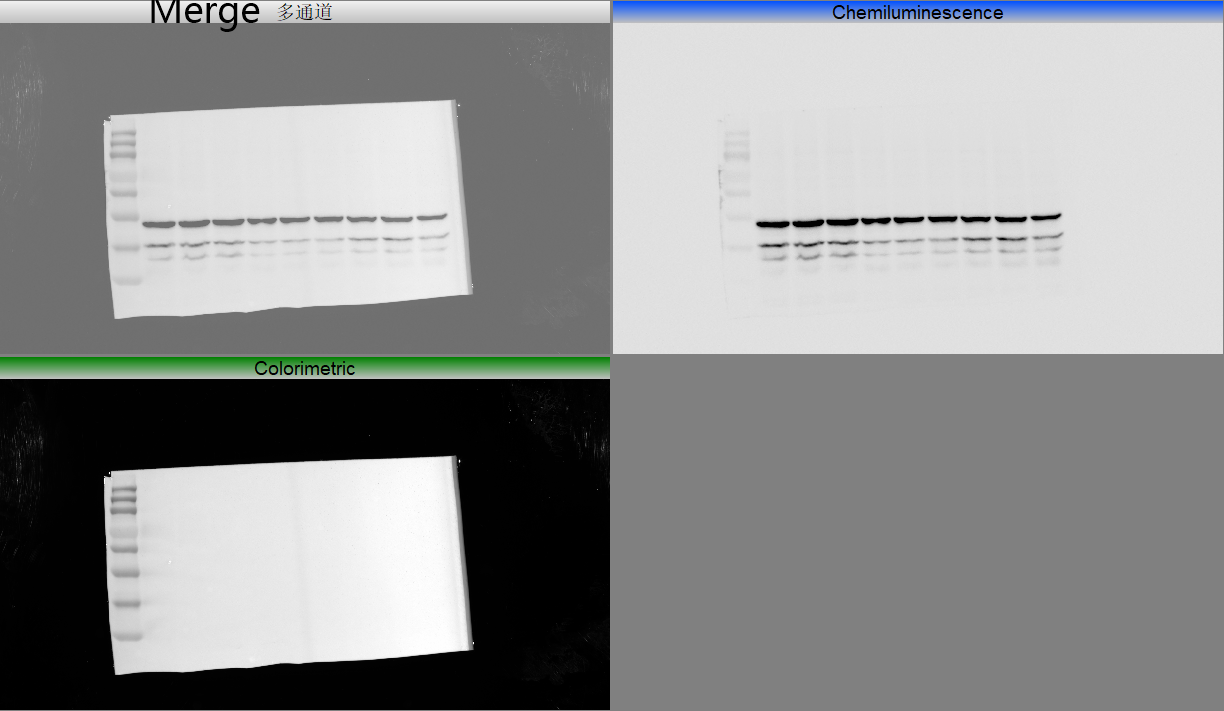

Supplement: Supplemental Information 2 — The raw data shows that the levels of BDNF expression and TrkB phosphorylation (p-TrkB) in the hippocampal region of the CLP mice were significantly reduced, relative to that in the sham group, but almost rescued in the LGG group of mice. [file peerj-12-17427-s002.zip › Supplementary files 2. (WB raw data)/BDNF2.tif]

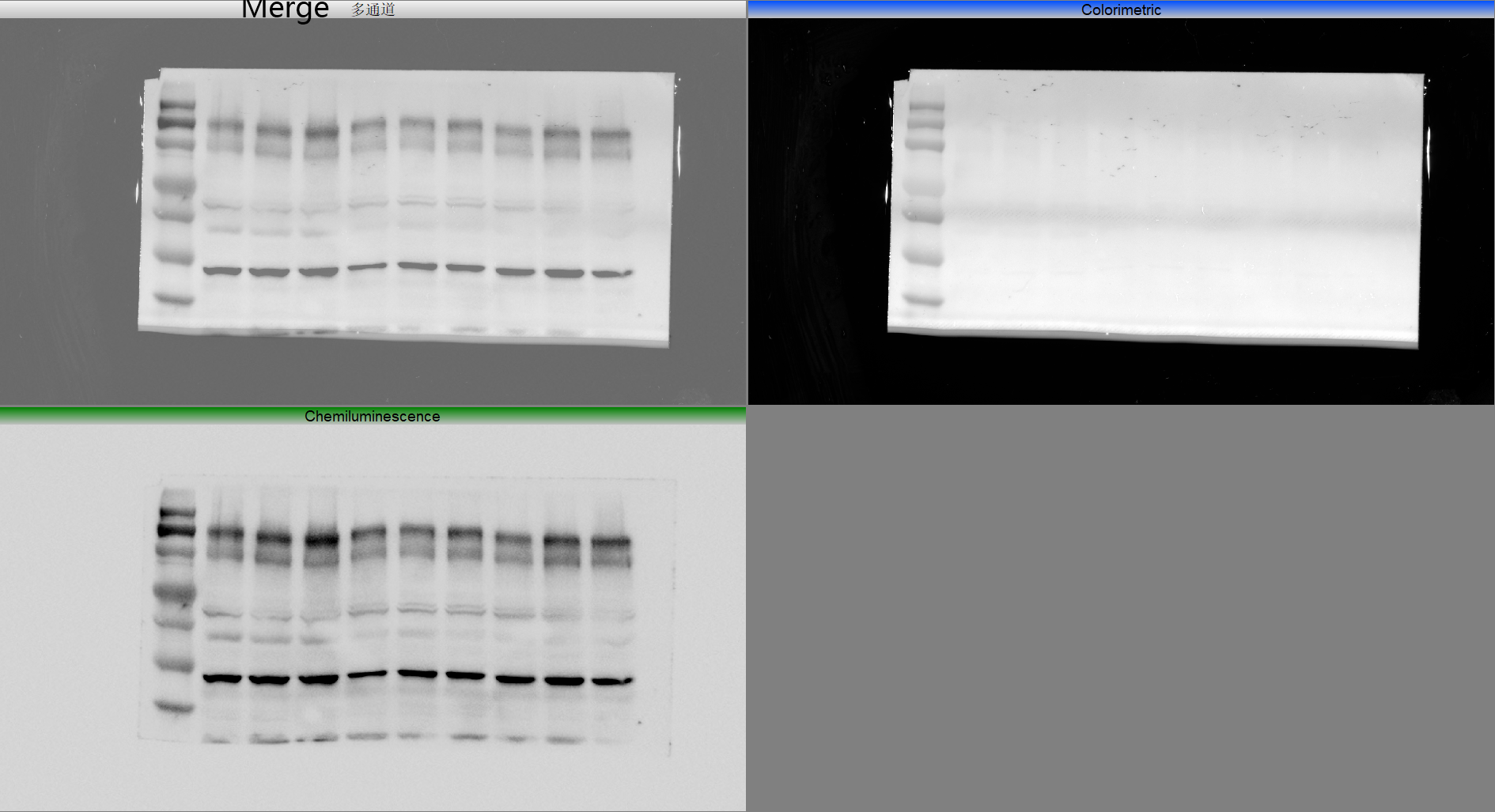

Supplement: Supplemental Information 2 — The raw data shows that the levels of BDNF expression and TrkB phosphorylation (p-TrkB) in the hippocampal region of the CLP mice were significantly reduced, relative to that in the sham group, but almost rescued in the LGG group of mice. [file peerj-12-17427-s002.zip › Supplementary files 2. (WB raw data)/BDNF3.tif]

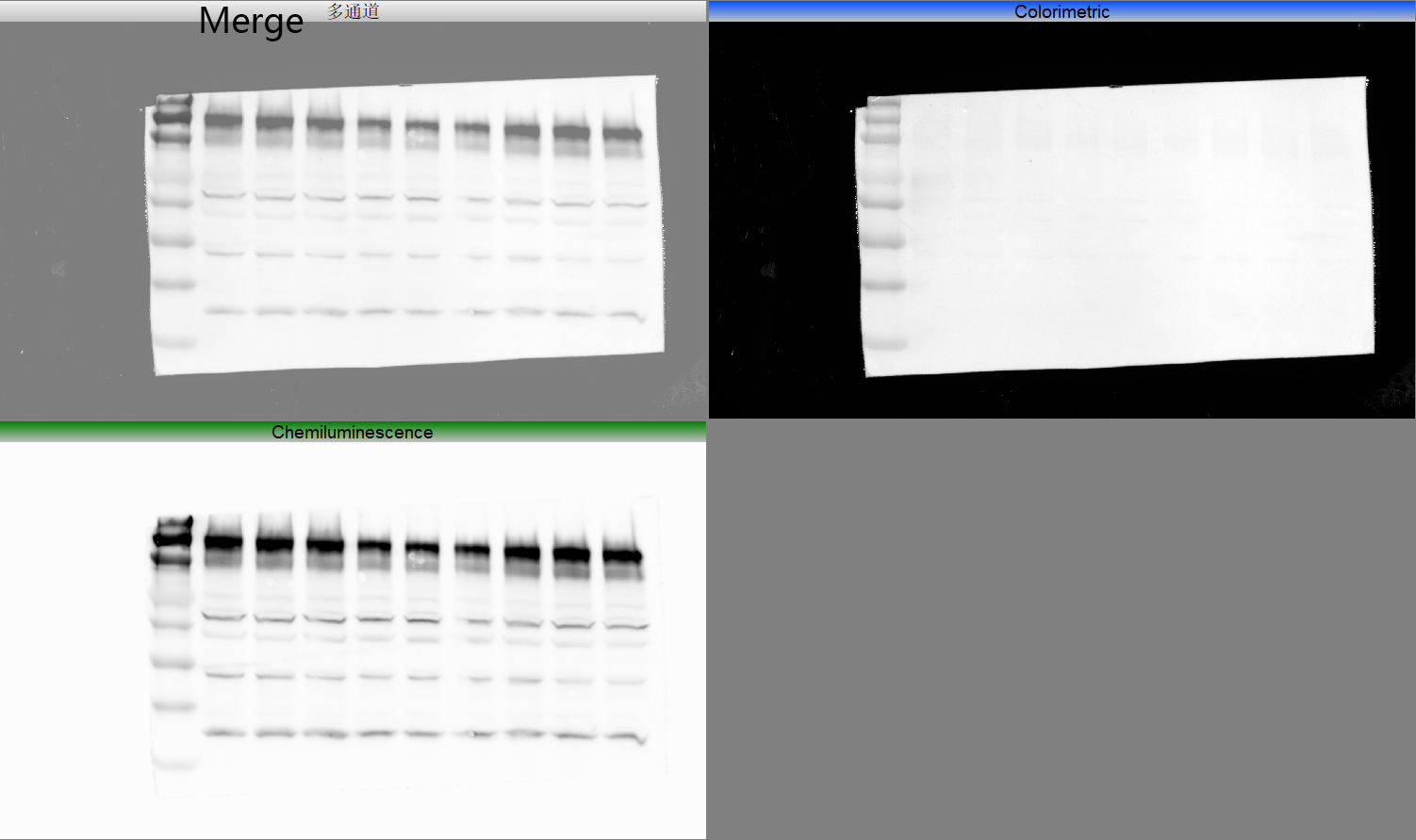

Supplement: Supplemental Information 2 — The raw data shows that the levels of BDNF expression and TrkB phosphorylation (p-TrkB) in the hippocampal region of the CLP mice were significantly reduced, relative to that in the sham group, but almost rescued in the LGG group of mice. [file peerj-12-17427-s002.zip › Supplementary files 2. (WB raw data)/P-TRKB2.tif]

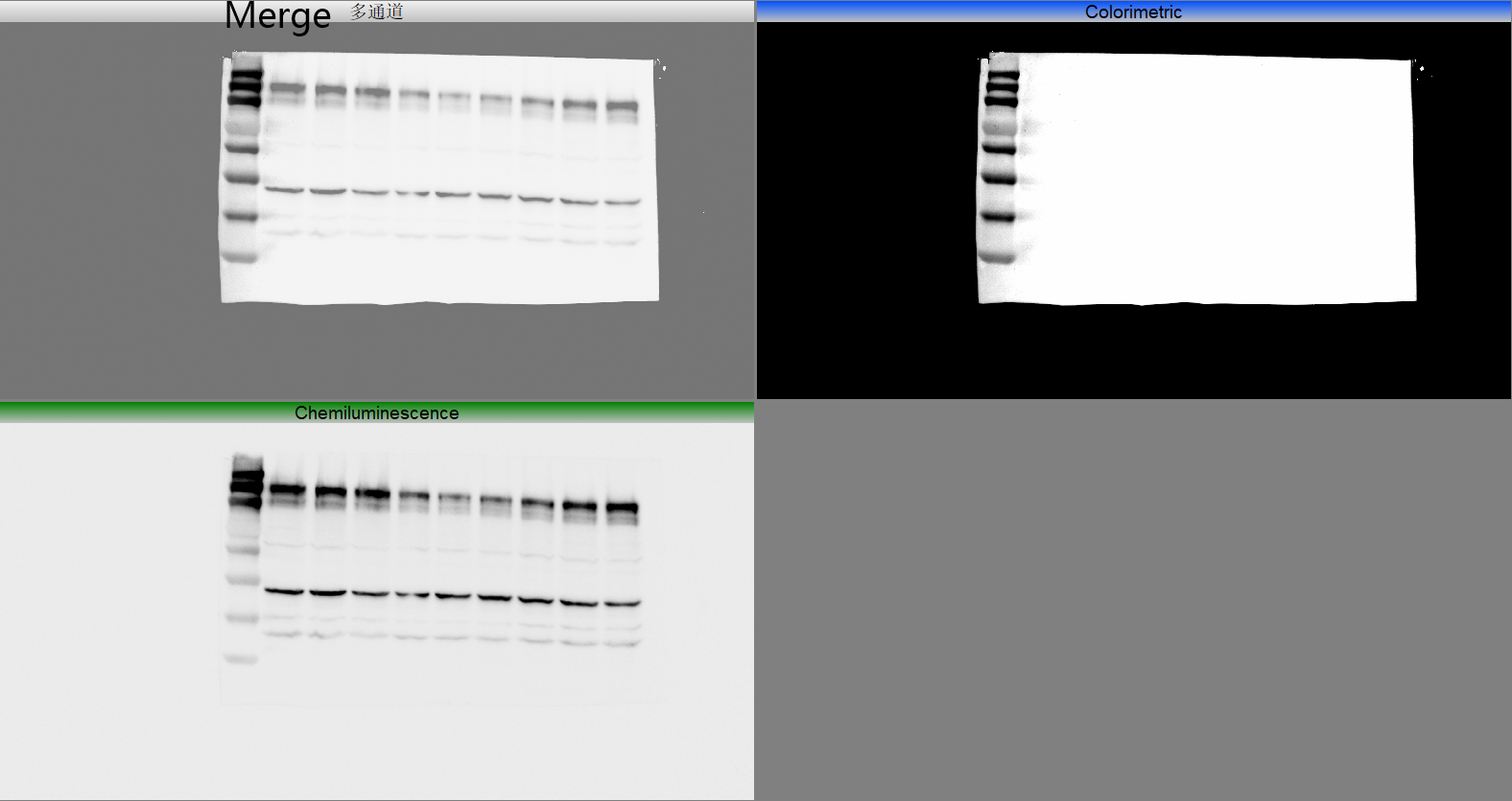

Supplement: Supplemental Information 2 — The raw data shows that the levels of BDNF expression and TrkB phosphorylation (p-TrkB) in the hippocampal region of the CLP mice were significantly reduced, relative to that in the sham group, but almost rescued in the LGG group of mice. [file peerj-12-17427-s002.zip › Supplementary files 2. (WB raw data)/P-TrkB1.tif]

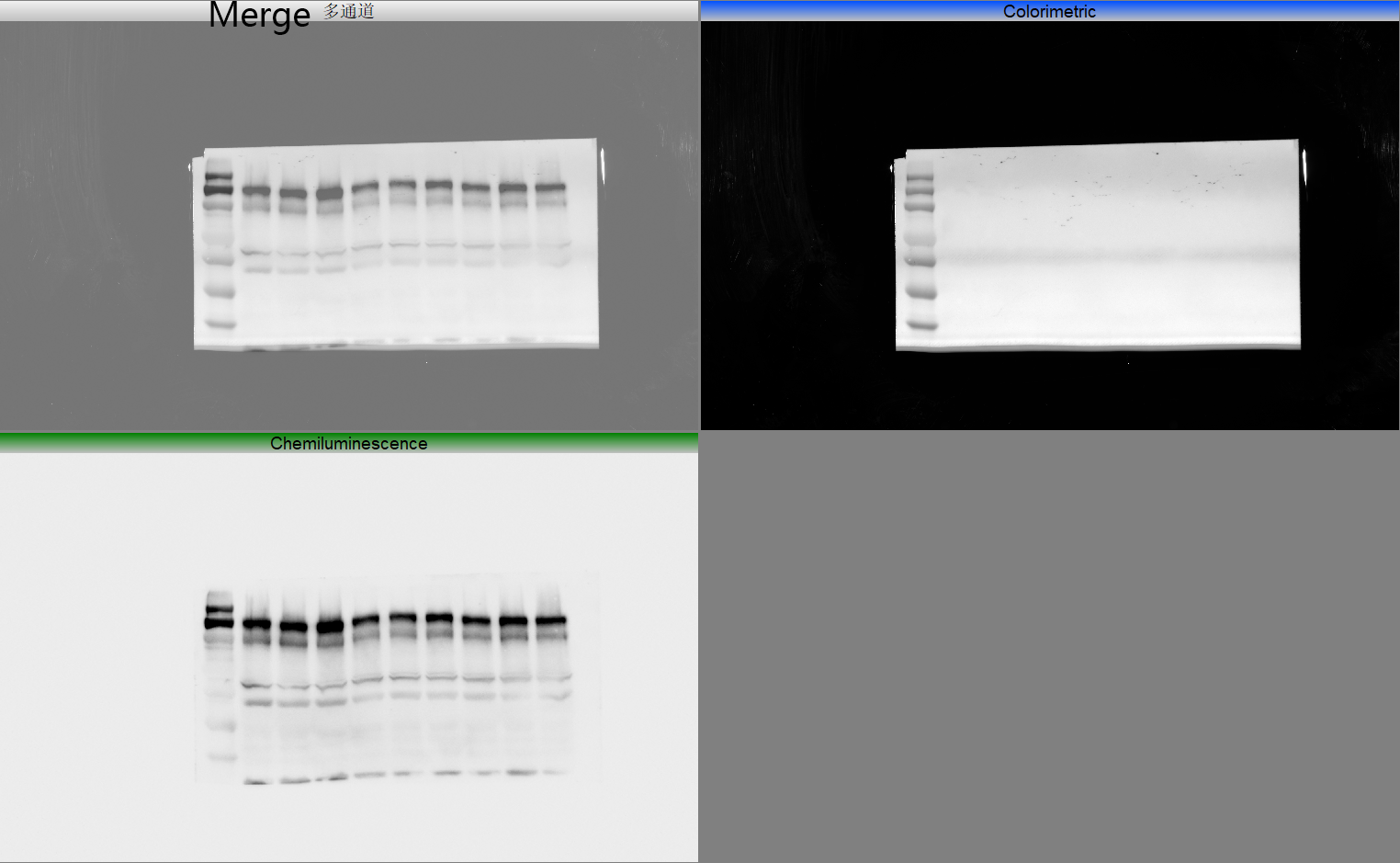

Supplement: Supplemental Information 2 — The raw data shows that the levels of BDNF expression and TrkB phosphorylation (p-TrkB) in the hippocampal region of the CLP mice were significantly reduced, relative to that in the sham group, but almost rescued in the LGG group of mice. [file peerj-12-17427-s002.zip › Supplementary files 2. (WB raw data)/P-TrkB3.tif]

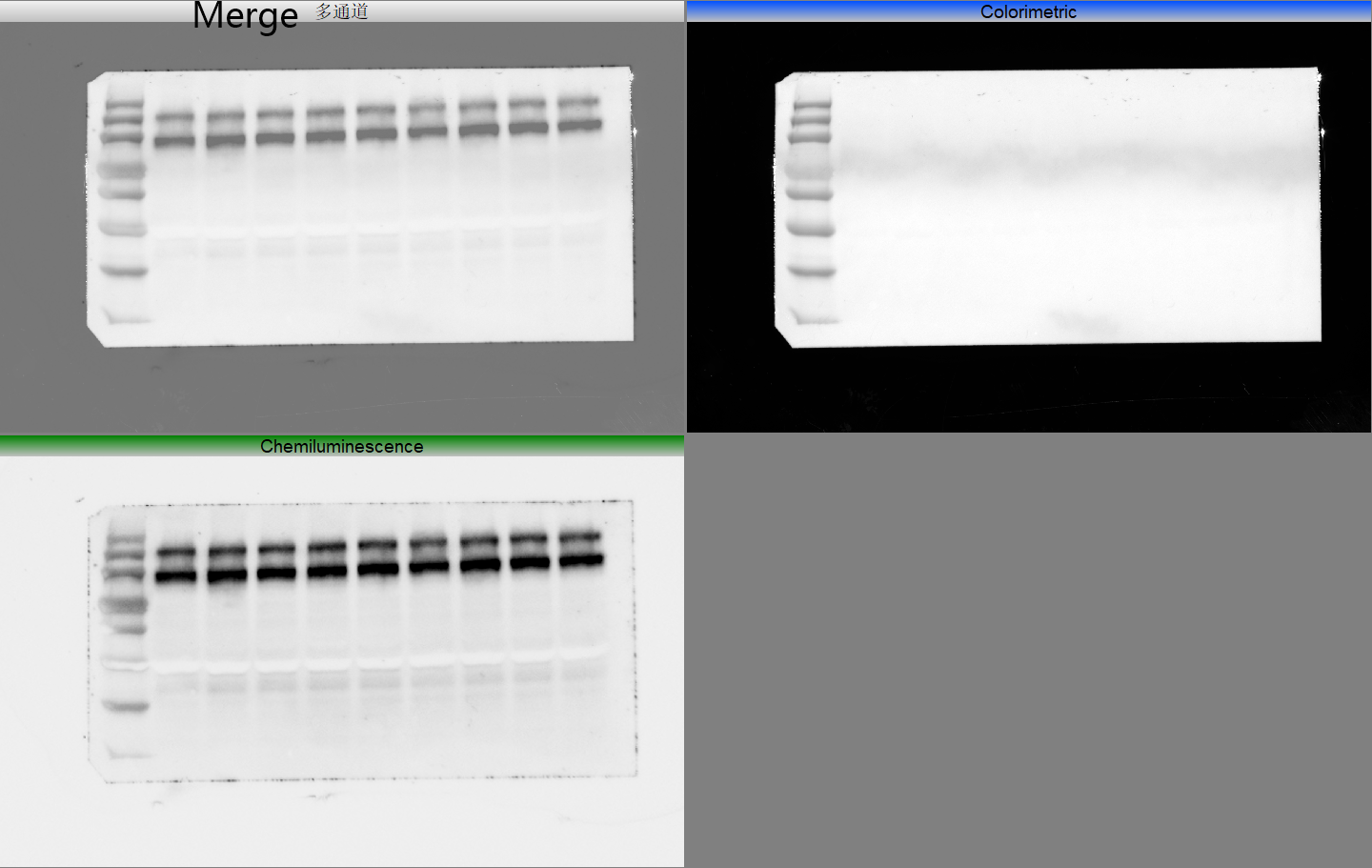

Supplement: Supplemental Information 2 — The raw data shows that the levels of BDNF expression and TrkB phosphorylation (p-TrkB) in the hippocampal region of the CLP mice were significantly reduced, relative to that in the sham group, but almost rescued in the LGG group of mice. [file peerj-12-17427-s002.zip › Supplementary files 2. (WB raw data)/TrkB 2.tif]

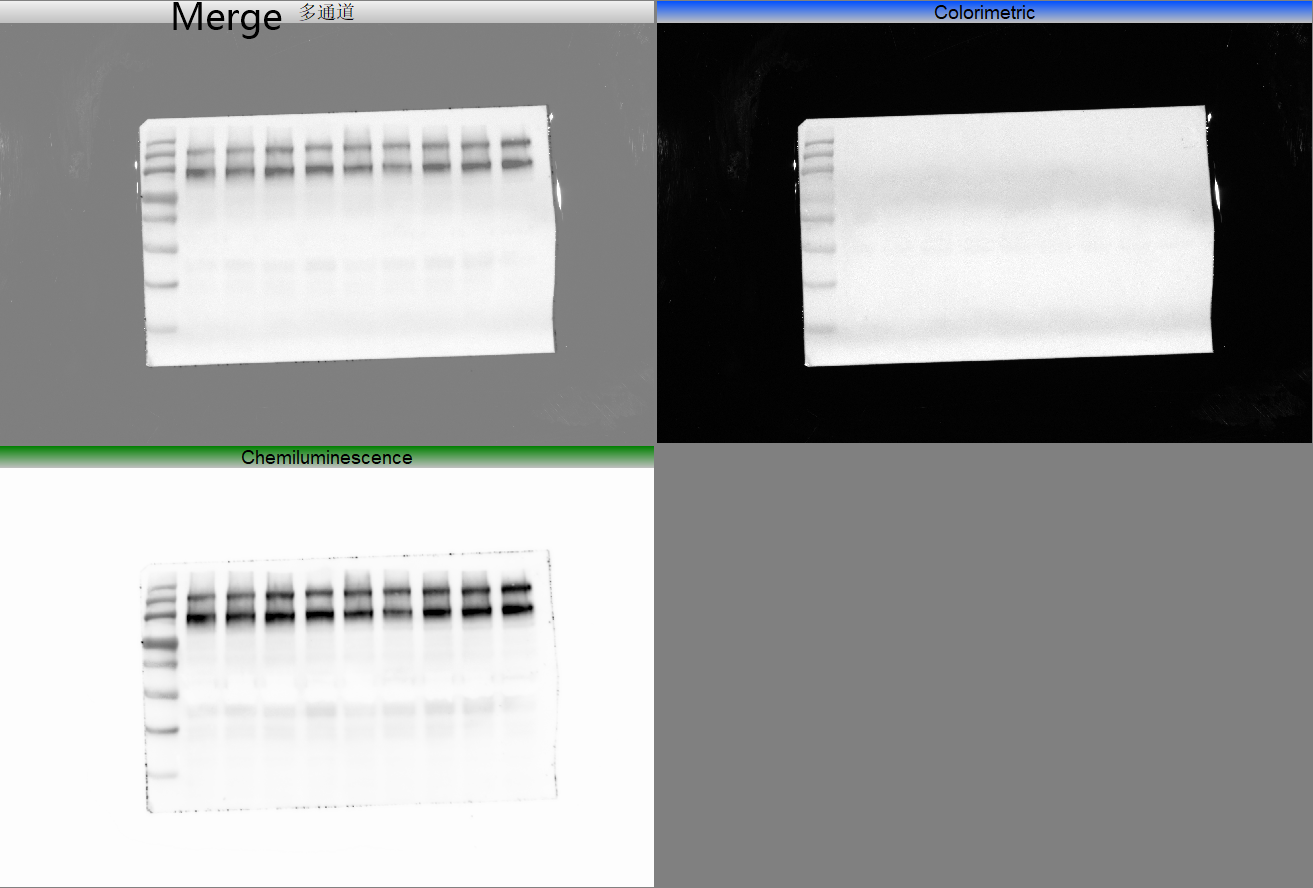

Supplement: Supplemental Information 2 — The raw data shows that the levels of BDNF expression and TrkB phosphorylation (p-TrkB) in the hippocampal region of the CLP mice were significantly reduced, relative to that in the sham group, but almost rescued in the LGG group of mice. [file peerj-12-17427-s002.zip › Supplementary files 2. (WB raw data)/TrkB 4.tif]

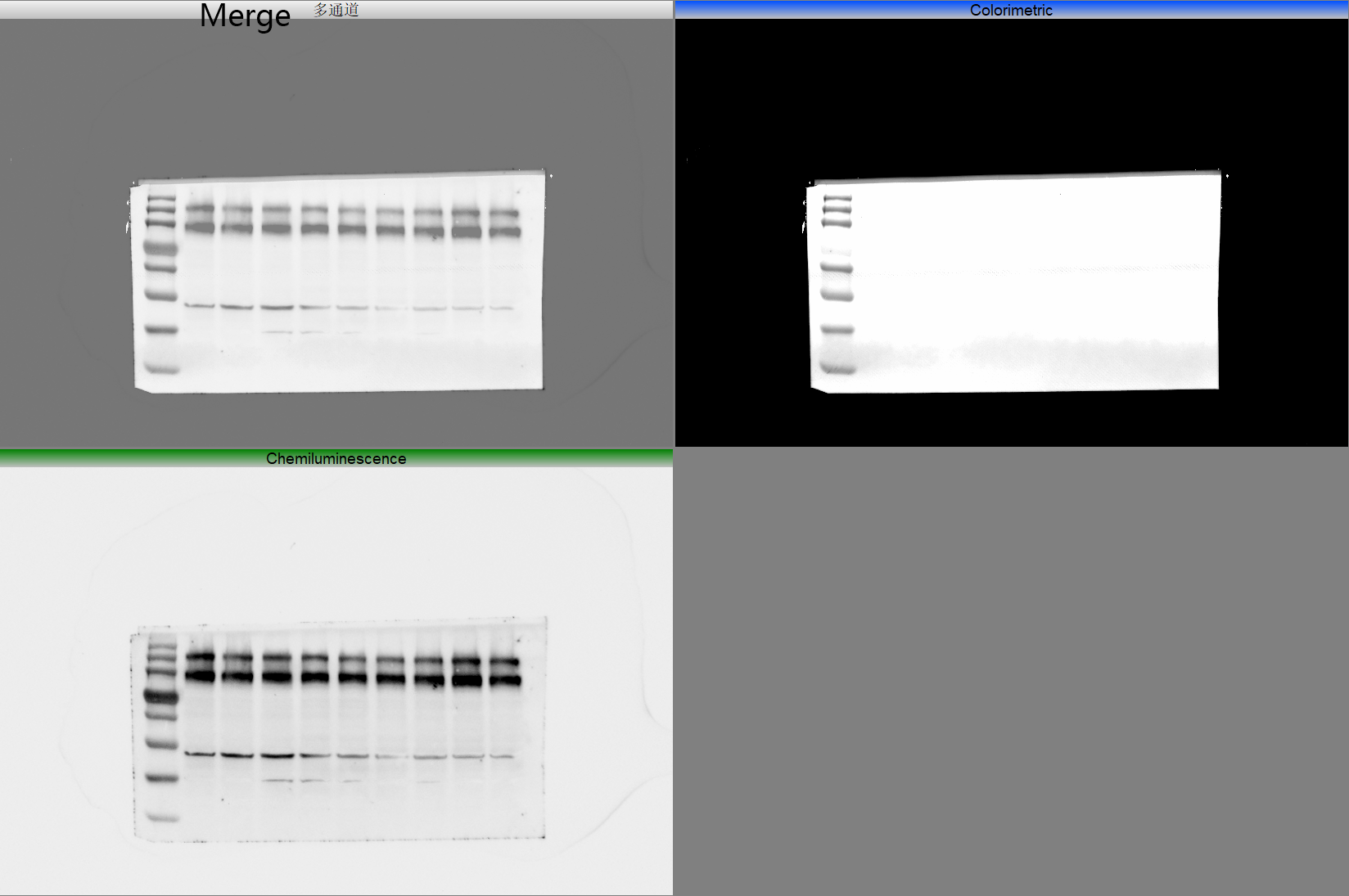

Supplement: Supplemental Information 2 — The raw data shows that the levels of BDNF expression and TrkB phosphorylation (p-TrkB) in the hippocampal region of the CLP mice were significantly reduced, relative to that in the sham group, but almost rescued in the LGG group of mice. [file peerj-12-17427-s002.zip › Supplementary files 2. (WB raw data)/TrkB1.tif]

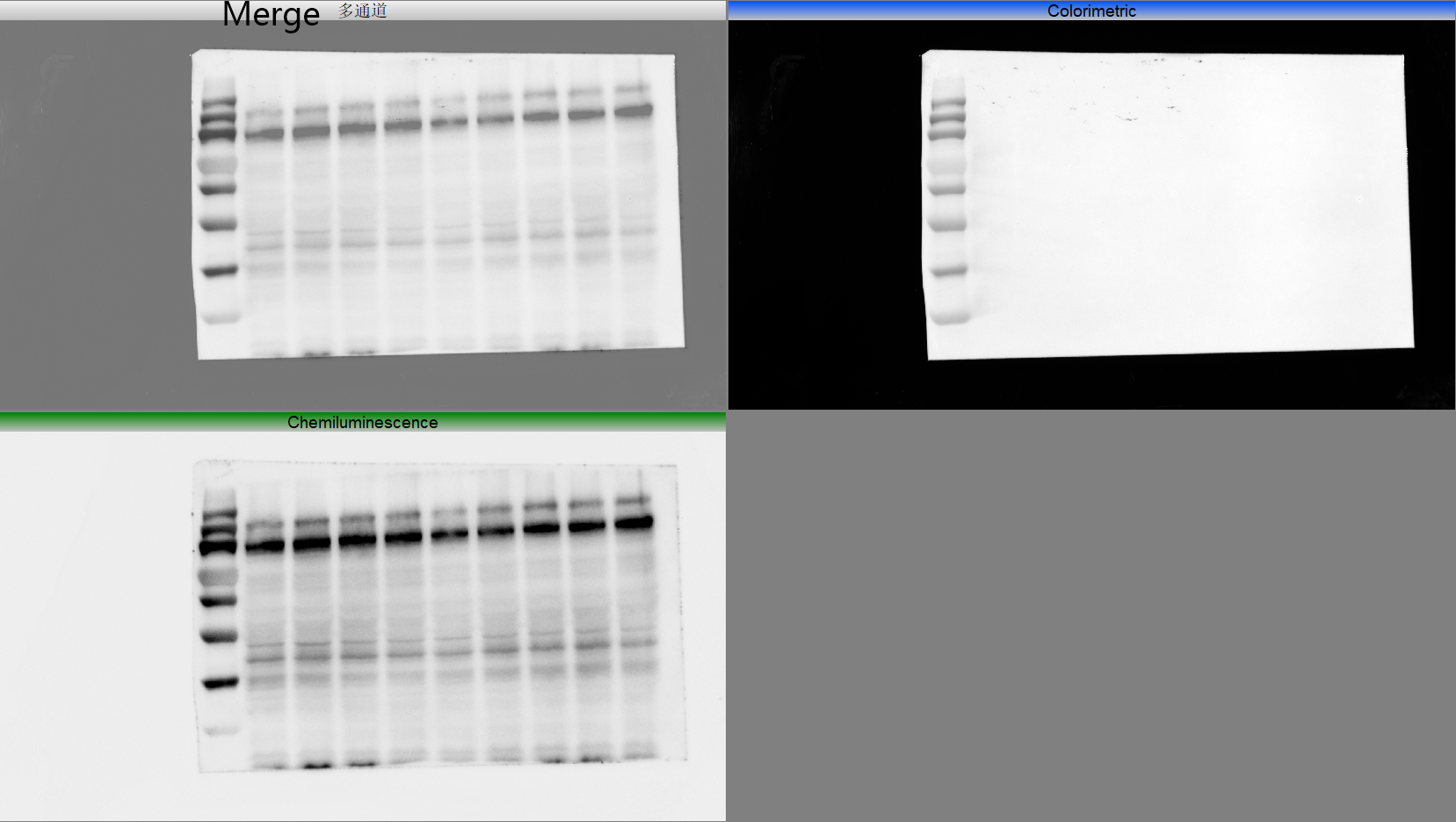

Supplement: Supplemental Information 2 — The raw data shows that the levels of BDNF expression and TrkB phosphorylation (p-TrkB) in the hippocampal region of the CLP mice were significantly reduced, relative to that in the sham group, but almost rescued in the LGG group of mice. [file peerj-12-17427-s002.zip › Supplementary files 2. (WB raw data)/TrkB3.tif]

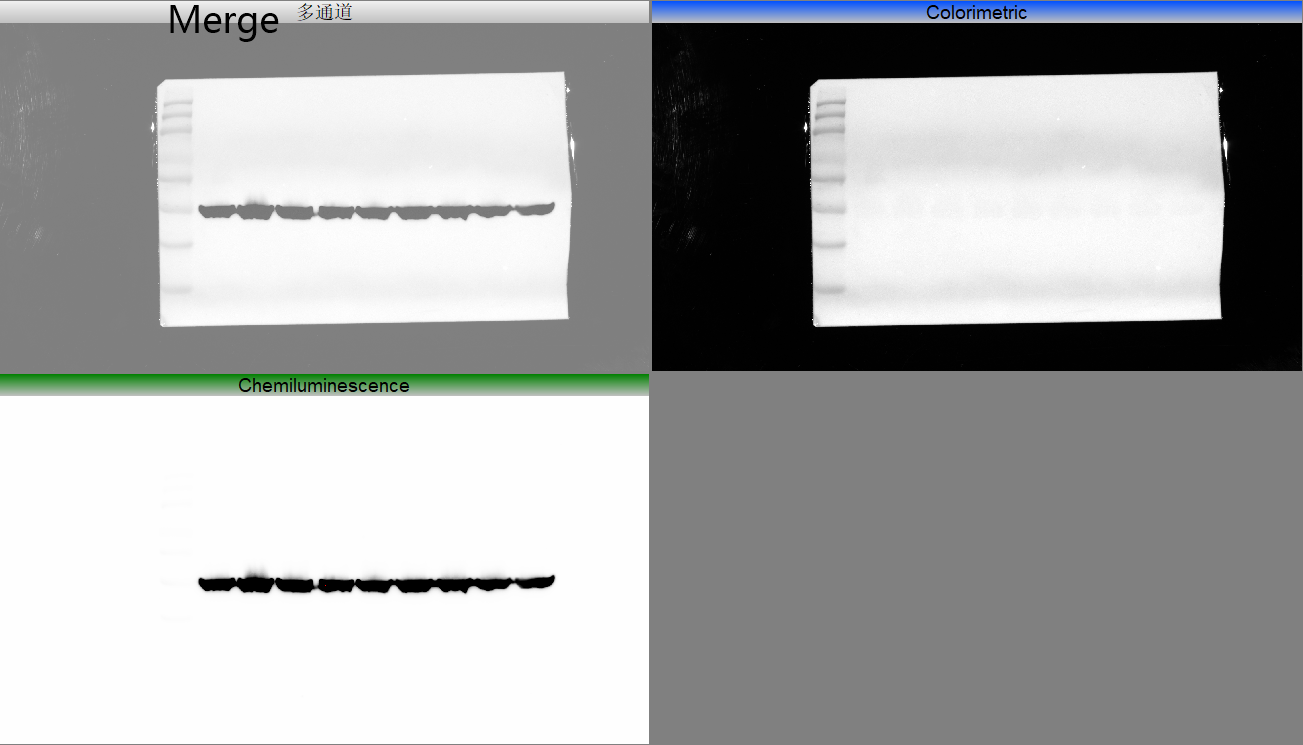

Supplement: Supplemental Information 2 — The raw data shows that the levels of BDNF expression and TrkB phosphorylation (p-TrkB) in the hippocampal region of the CLP mice were significantly reduced, relative to that in the sham group, but almost rescued in the LGG group of mice. [file peerj-12-17427-s002.zip › Supplementary files 2. (WB raw data)/β-actin1.tif]

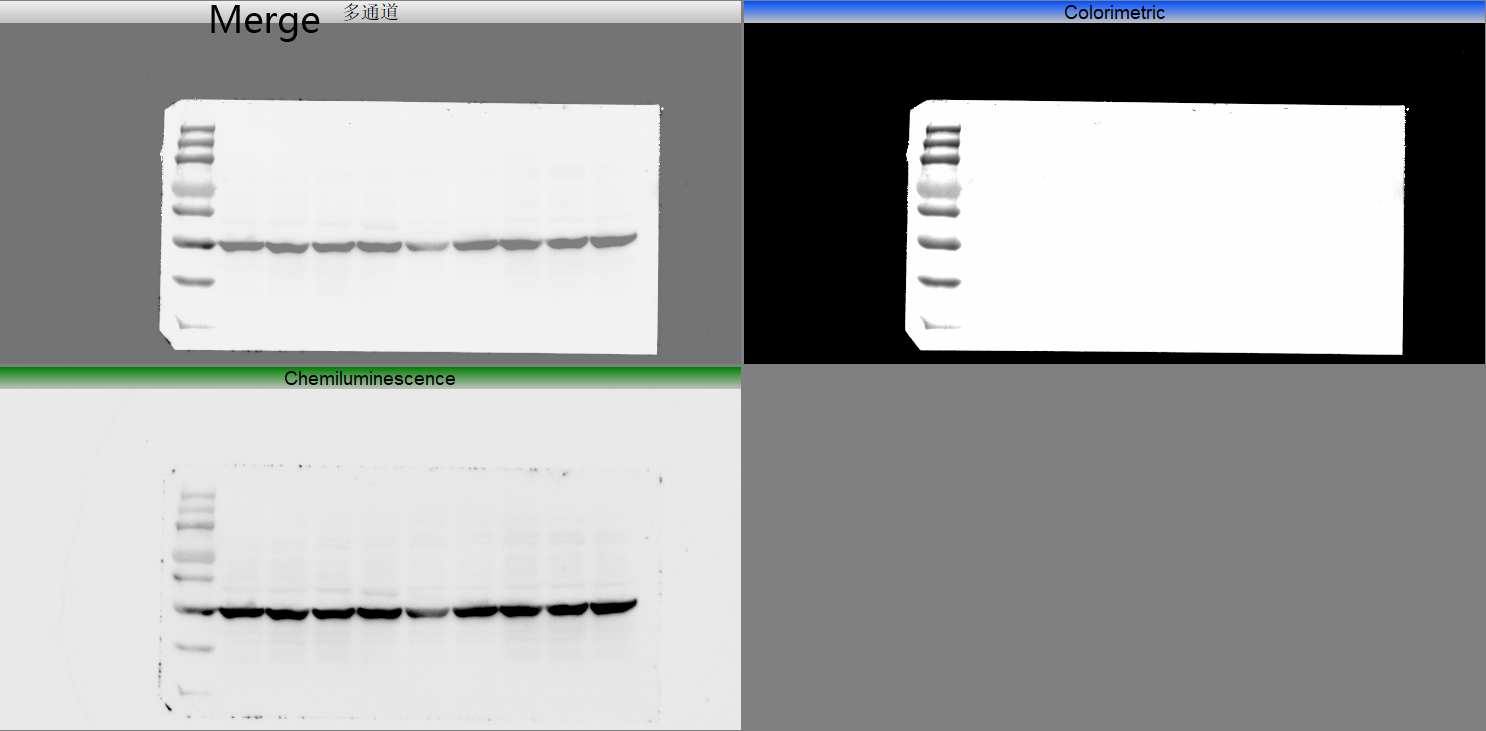

Supplement: Supplemental Information 2 — The raw data shows that the levels of BDNF expression and TrkB phosphorylation (p-TrkB) in the hippocampal region of the CLP mice were significantly reduced, relative to that in the sham group, but almost rescued in the LGG group of mice. [file peerj-12-17427-s002.zip › Supplementary files 2. (WB raw data)/β-actin2.tif]

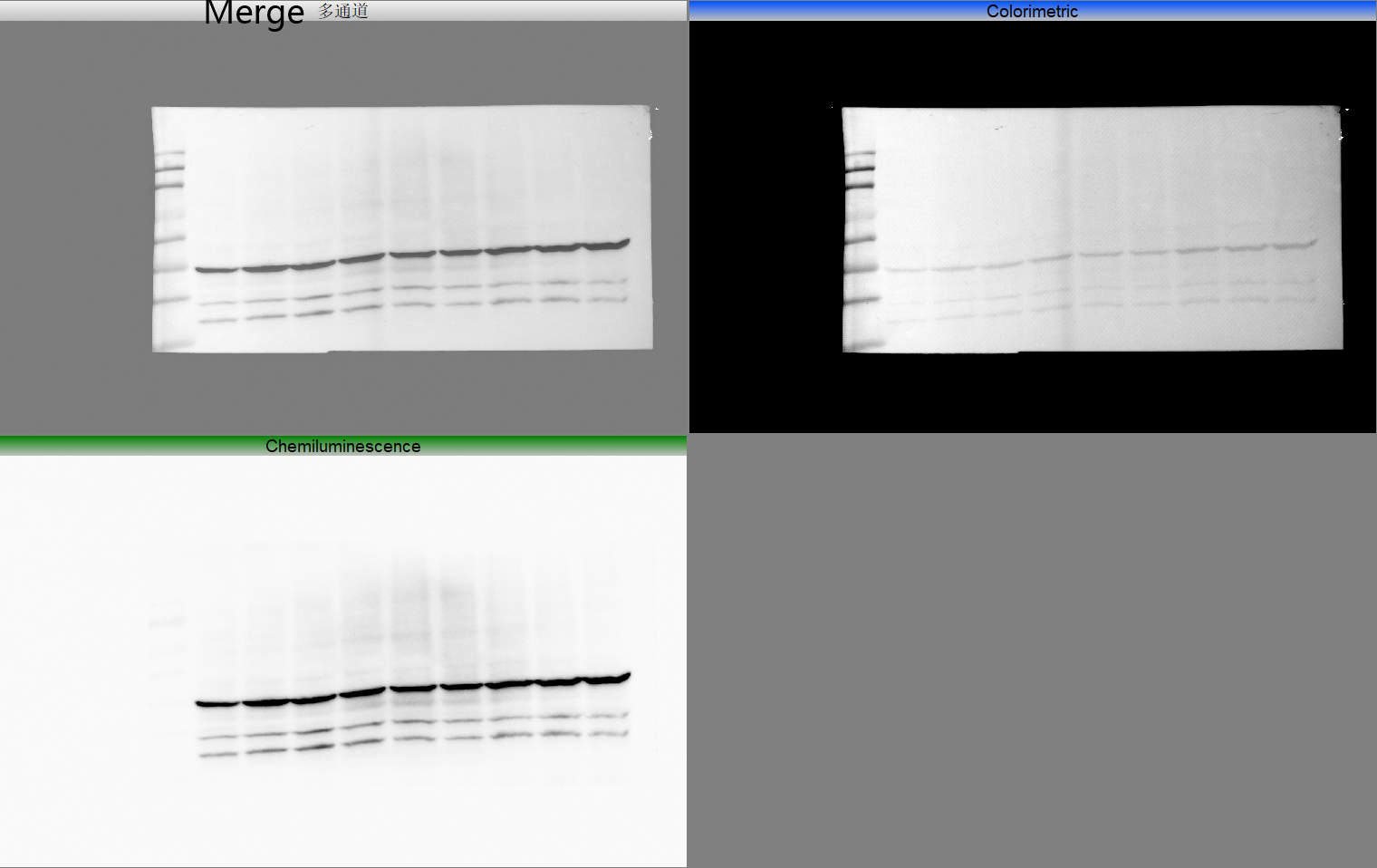

Supplement: Supplemental Information 2 — The raw data shows that the levels of BDNF expression and TrkB phosphorylation (p-TrkB) in the hippocampal region of the CLP mice were significantly reduced, relative to that in the sham group, but almost rescued in the LGG group of mice. [file peerj-12-17427-s002.zip › Supplementary files 2. (WB raw data)/β-actin3.tif]
